# Chronic Pain after Open Appendectomy and Its Effects on Quality of Life in Children Aged 8–18 Years

**DOI:** 10.1155/2021/6643714

**Published:** 2021-02-22

**Authors:** Onur Palabiyik, Gurkan Demir

**Affiliations:** Sakarya University, Faculty of Medicine, Department of Anesthesiology and Reanimation, Sakarya, Turkey

## Abstract

**Background:**

Chronic postsurgical pain is an important problem for both children and adults. This study aims to investigate the prevalence of chronic postappendectomy pain (CPAP) in children and its social and physical effects.

**Methods:**

This prospective observational study was conducted on children aged 8–18 years who had undergone open appendectomies. In the sixth month after the surgical procedure, the presence of chronic pain was examined in the lower right abdominal area. CPAP and its effects on children's daily life activities were assessed using the numeric rating scale (NRS) and the Pediatric Quality of Life Scale (PedsQL).

**Results:**

Analysis was performed on 158 children, 97 of whom were boys (61.4%) and 61 were girls (38.6%). The average age was 12.8 ± 3 years, the average NRS was 4.48 ± 1.1, and the average scar length was 6.09 ± 1.6 cm. Twenty-nine children described CPAP, and its prevalence at six months after the surgery was 18.4%. Of these, 16 (55.2%) complained of pain only during exercise and 13 (44.8%) experienced pain while resting. The rate of CPAP was significantly higher in girls. Female gender and longer scar length were associated with the development of chronic pain. The PedsQL scores from the children's self-reports and their parents' reports were significantly lower for children who described CPAP as compared to those without CPAP.

**Conclusion:**

CPAP occurs quite frequently in children, especially in girls, and negatively affects children's quality of life.

## 1. Introduction

The treatment of acute appendicitis is an appendectomy, and it is the most common emergency surgical procedure in children [1–3]. An appendectomy is performed as open or laparoscopic surgery in both children and adults [4–6]. Laparoscopic appendectomy is performed more often and more commonly than open surgery [6, 7]; nevertheless, surgeons choose between open and laparoscopic appendectomy according to their beliefs about the clinical benefits and complications of both techniques [4]. Although laparoscopic appendectomy is prominent in terms of its clinical benefits, including reduced cardiovascular and pulmonary complications, shortened hospital stay, lesser postoperative pain, earlier recovery, outstanding cosmetic results, an earlier return of intestinal function, reduced adhesion formation, and lower rates of wound infection [4, 5, 8], there are still centres where open appendectomy is performed more frequently [6, 7].

Chronic postsurgical pain (CPSP) is defined as pain that develops after surgery and lasts at least two months [9]. Considering the large number of surgical procedures performed in children, little is known about the prevalence of chronic postoperative pain in children and its effects on their daily life activities as compared to adults.

Some paediatric patients who had undergone open appendectomy complained of lower right abdominal pain in the postoperative period; this complaint that may affect the quality of life should not be ignored. Because we did not find this topic in the literature, we aimed to investigate the prevalence of postoperative chronic lower right abdominal pain in children aged 8–18 years who had undergone open appendectomy and its social and physical effects.

## 2. Materials and Methods

### 2.1. Patient Selection

This study was registered at ClinicalTrials.gov, with the identifier NCT03791229. Following a local university ethics committee's approval (2018-E.14113), all children who underwent appendectomies between May 2018 and April 2019 were included in this prospective observational study. Their records, including age, gender, American Society of Anesthesiology (ASA) classification score, and type of surgical procedure, were obtained from the hospital information management system and anaesthetic charts. Children aged 8–18 years were included in this study. Those with physical scores of ASA III or higher, preoperative pain complaints over the six month period, and psychiatric disorders, along with those who had previously undergone surgery on the lower right abdomen, were excluded from this study. Additionally, children who underwent laparoscopic appendectomies were excluded.

Pediatric surgeons performed surgical procedures according to their knowledge and beliefs, and all open appendectomies were performed as reported in the literature [7]. In our routine practice, intravenous 10 mg/kg of acetaminophen was administered intraoperatively and then every 6–8 hours postoperatively to provide postoperative analgesia.

The primary aim of this study was to evaluate chronic lower right abdominal pain at six months after open appendectomy, which was defined as CPAP, and to determine its prevalence. Our secondary outcome was to assess the effects of pain on the quality of life in children. The primary endpoint of this study was to reach the number of children forming the sample size calculated with a statistical program.

### 2.2. Evaluation of Pain

In the sixth month after the surgical procedure, the children and their parents were contacted by telephone, and those who provided informed consent were asked if they had chronic pain in the surgery area. The children were asked whether they felt pain while resting and exercising. Pain was defined as the presence of an intermittent or continuous hurtful sensation. In this study, CPAP in the lower right abdominal area was assessed using the verbal numeric rating scale (NRS). When the NRS scored 3 and above, it was defined as CPAP. The social and physical effects of chronic pain were also assessed using the Pediatric Quality of Life Scale (PedsQL).

The NRS is a commonly used tool for measuring pain intensity in clinical practice [10]. Patients are asked to score pain intensity numerically from 0 to 10. A score of 0 indicates that the patient has no pain, and a score of 10 indicates that the patient has the worst imaginable pain.

The PedsQL is a questionnaire developed by Varni et al. [11] to measure the health-related quality of life of children aged 2–18 years. The PedsQL questions the areas of physical health, emotional functioning, and social functioning to determine the child's health status and state at school. Items are scored between 0 and 100. The answer is 100 if it is never marked, 75 if it is rarely marked, 50 if it is sometimes marked, 25 if it is often marked, and 0 if it is almost always marked [12]. Higher total PedsQL scores are associated with better health-related quality of life [13].

The children's scar lengths were measured by their parents. How to measure the scar length of the children was explained to their parents over the phone. These data were obtained from interviews with their parents.

### 2.3. Statistical Analysis

Our primary outcome was the prevalence of CPAP. To determine the sample size, a pilot study was conducted. Ten children were evaluated, and CPAP was detected in two of them. Assuming that the prevalence of CPSP in the lower abdominal area was approximately 10% [14, 15], 155 children were required to achieve a statistical power of 95% and alpha error of 0.05 to detect a twofold higher prevalence of CPAP in the lower right abdominal area at six months after open appendectomy.

All statistical analyses were performed using the Statistical Package for the Social Sciences 25 for Windows. All data are expressed as a number, minimum, maximum, percent, and mean and standard deviation were appropriate. The numerical data for the two groups were compared using Student's *t*-test. Associations between the categorical variables were analysed using Pearson's chi-squared and Fisher's exact tests. A logistic regression analysis was performed to identify the relationship between the variables and chronic pain. A value of *p* < 0.05 was considered statistically significant.

## 3. Results

**Figure 1:**
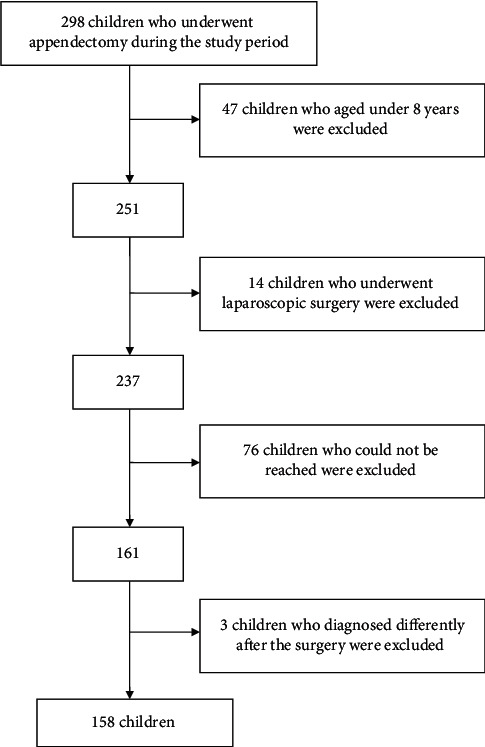
Flow chart.

During the study period, 298 children underwent an appendectomy at our hospital. Of those, 47 were less than 8 years old, 14 underwent a laparoscopic appendectomy, 76 could not be reached by any method, and 3 received a different diagnosis after the surgery and were excluded from the study (Figure 1). Ultimately, 158 children were enrolled in the study: 97 boys (61.4%) and 61 girls (38.6%) (Table 1).

The mean age of the children was 12.8 ± 3 (8–18) years. The mean scar length was 6.09 ± 1.6 (2.2–9.8) cm (Table 1). The mean NRS was 4.48 ± 1.1 (3–6). Twenty-nine of the 158 children described CPAP in the lower right abdominal area six months after the surgery. The prevalence of CPAP at six months after the surgery was 18.4%. Of the children with CPAP, 16 (55.2%) complained of pain only during exercise and 13 (44.8%) experienced pain while resting (Table 1). Fifteen (51.7%) of those children described their pain as stinging, eight (27.6%) as blunt, four (13.8%) as cramping, and two (6.9%) as burning (Table 1). The mean ages of children with and without CPAP were 13.24 ± 2.7 and 12.70 ± 3.1 years, respectively, and were similar to each other (*p*=0.387) (Table 2). The mean NRS scores reported by the children aged 8–12 years and 13–18 years were 4.92 ± 1.0 and 4.13 ± 1.0, respectively, and the mean score was significantly higher in children aged 8–12 years (*p*=0.041). The mean NRS scores reported by boys and girls were 4.75 ± 1.0 and 4.30 ± 1.1, respectively. These were similar (*p*=0.260). The rate of CPAP was significantly higher in girls as compared to boys (*p*=0.014) (Table 2).

According to logistic regression analysis, no relationship was found between age and chronic pain (*p*=0.144), whereas gender difference and scar length were significantly associated with chronic pain (*p*=0.016 and *p* < 0.001, respectively) (Table 3).

The PedsQL scores from the children's self-reports and their parents' reports were significantly lower for children who described CPAP as compared to those without CPAP (*p* < 0.001, *p*=0.025, and *p*=0.009) (Table 4).

## 4. Discussion

In our study, we found that in children, the prevalence of CPAP in the lower right abdominal area at six months following open appendectomy was 18.4%. CPAP was more common in girls. The female gender and longer scar length were associated with the development of chronic pain. Besides, CPAP was negatively affecting the children's daily life activities.

CPSP is an important problem for both children and adults. Studies have reported the prevalence of CPSP in children as varying between 5% and 54% [15, 16]. The reasons for this variability are the evaluations of different surgical procedures and pain assessments performed at different times after surgery. With regard to the chronic lower right abdominal pain, there are some data only following inguinal hernia repair procedures. In one study, the rate of CPSP in the lower right abdominal area following inguinal hernia repair procedures in children was 7.1% at 12 months and 5.1% at three years after surgery [15]. Mossetti et al. [14] reported that the rates of CPSP at one, three, and six months after inguinal herniorrhaphy were 35.6%, 14.9%, and 9.2%, respectively. A study that included children under five years of age reported that, while the prevalence of CPSP was 13.5%, moderate or severe pain was observed in only 2% of the children [17]. There are no reports in the literature regarding chronic lower right abdominal pain after appendectomy. In our study, the prevalence of CPAP in the lower right abdominal area at six months after open appendectomy in children was 18.4%. Our opinion is that one of the reasons why this rate is higher than other studies is due to the long scar size. The presence of preoperative abdominal pain, which has been reported as a risk factor for chronic pain development [9, 16, 18], in children may have contributed to this high prevalence. Besides, the anxiety associated with acute surgical procedure in both children and their parents in the preoperative period should not be ignored. This emotional situation increases the prevalence of chronic pain [9, 16, 18].

The prevalence of CPSP is lower in children than in adults [9, 18]; however, CPSP increases in frequency as the age of the children increases [15, 19, 20]. The higher CPSP prevalence in adults may involve psychological factors, such as fear of surgery, whereas children are mostly not hesitant about surgery [21]. The parents' cognitions and behaviours related to their children's pain directly affect their symptom experiences and may affect their children's perceptions of pain and cognition [16, 22–24]. Kristensen et al. [15] showed that the risk for the development of CPSP in childhood increases with age. In contrast, another study found no significant correlation between CPSP and child age in children aged younger than three months when the surgical timing was evaluated [17]. Several studies reported no significant relation between CPAP and child age [14, 18, 25]. In our study, a significant relationship was not found between the age of children and the development of chronic pain. However, among children with chronic pain, NRS scores were reported as significantly higher in the children aged 8–12 than in the children aged 13–18.

In adults, the CPSP is more common in females than in males [26]. Contradictory results have been reported on whether gender is important in the development of CPSP in children. One study reported no significant difference between genders in CPSP at six and 12 months after surgery [27]. In another study, no differences were observed between genders in the development of CPSP [25]. However, in several studies, higher chronic pain scores and prevalence were reported in girls [28–30]. Although the mean points of NRS reported by girls and boys in our study were similar, the CPAP was more common in girls who were also more likely to develop chronic pain.

Although the development mechanisms of CPSP have not yet been fully explained, it is known that nerve damage plays an important role. Nerve damage can occur mainly due to reasons such as incision and traction during the surgery. Nerve damage can also occur due to excessive fibrosis, squeezing, and kinking during the scar formation in the incision area in the postoperative period. For this reason, the surgical procedures that reduce nerve damage should be applied. Batoz et al. [18] reported that the scar size is a risk factor for the development of chronic pain. In our study, the scar length was longer in children who described CPAP than in children without chronic pain.

Chronic pain in children is associated with poorer health outcomes and greater functional disability after surgery [14, 25, 31, 32]. Roth-Isigkeit et al. [19] showed that quality of life and restrictions in daily activities are related to intensity of pain and that intensity of pain is the strongest variable for predicting impairment in various areas of life. Our study shows that the children who described pain had more restrictions in daily life and life as compared to the children who described no chronic pain.

This means that one of the most important factors in the development of CPSP is the type of surgery. Our study was originally planned to examine all cases undergoing open and laparoscopic appendectomy and evaluate the prevalence of CPAP. However, since laparoscopic appendectomies were too few to compare during the study period, these cases were excluded from the study and only open appendectomy cases were evaluated. We consider this the most important limitation of our study. Another limitation is that our study is a single-centre experience. Furthermore, we have no data, such as preoperative and postoperative acute pain scores, in our study due to it being focused on determining the prevalence of CPAP. Despite these limitations, our study includes a unique feature, as it is the first study on the prevalence of chronic pain after appendectomy, which is a common surgery in the paediatric population, and has a prospective design. Our study throws a new light on the consideration of a significantly neglected research area in paediatric populations.

As a consequence, chronic lower right abdominal pain after open appendectomy is seen more frequently than expected in paediatric patients, especially in girls. It should be kept in mind that developing techniques to reduce nerve damage in the surgical field and minimising skin incisions will be effective in reducing the development of chronic pain.

## Figures and Tables

**Table 1:** Demographic data of children.

	All children (*n* = 158)	Children with CPAP (*n* = 27)
Age (year)	12.80 ± 3.0 (8–18)	13.24 ± 2.7 (9–18)
Scar length (cm)	6.09 ± 1.6 (2.2–9.8)	7.05 ± 1.4 (3.7–9.3)
Gender		
Boy	97 (61.4%)	12 (41.4%)
Girl	61 (38.6%)	17 (58.6%)
Pain interference		
Resting		13 (44.8%)
Exercise		16 (55.2%)
Pain description		
Stinging		15 (51.7%)
Blunt		8 (27.6%)
Cramping		4 (13.8%)
Burning		2 (6.9%)

CPAP, chronic postappendectomy pain; *n*, number of children; %, percentage. Data are shown as mean ± SD, minimum, maximum, number, and percentage.

**Table 2:** Children characteristics according to chronic postappendectomy pain.

	Children without CPAP	Children with CPAP	*p*
Age	12.70 ± 3.1	13.24 ± 2.7	0.387
Scar length	5.87 ± 1.6	7.05 ± 1.4	<0.001^*∗*^
Gender			
Boy/girl	85/44	12/17	0.014^*∗*^

CPAP, chronic postappendectomy pain; *p*, statistical error. ^*∗*^Statistical significant. The data are presented number and mean ± SD.

**Table 3:** Logistic regression analysis for chronic pain.

	*β*	SE (*β*)	OR	%95 CI for OR (lower-upper)	*p*
Age	–0.132	0.091	0.876	(0.734–1.046)	0.144
Gender (male)	1.085	0.448	2.960	(1.229–7.128)	0.016^*∗*^
Scar length	0.677	0.189	1.968	(1.360–2.849)	<0.001^*∗*^
Constant	–4.669	1.219	0.009		0.000

*β*, regression coefficient; SE, standard error; OR, odds ratio; CI, confidence interval; *p*, statistical error. ^*∗*^Statistical significant.

**Table 4:** The comparison of the PedsQL scores of children.

PedsQL scores	Children without CPAP	Children with CPAP	*p*
Total score of PedsQL of child self-report	96.67 ± 3.9	75.11 ± 16.6	<0.001^*∗*^
Total score of PedsQL of child's parent report	97.42 ± 3.6	74.85 ± 17.4	<0.001^*∗*^
Physical score of PedsQL of child self-report	97.21 ± 4.6	68.62 ± 23.6	<0.001^*∗*^
Physical score of PedsQL of child's parent report	97.72 ± 4.6	69.16 ± 23.8	<0.001^*∗*^
Emotional score of PedsQL of child self-report	95.35 ± 5.9	68.10 ± 26.5	<0.001^*∗*^
Emotional score of PedsQL of child's parent report	97.05 ± 5.5	64.48 ± 27.3	<0.001^*∗*^
Social score of PedsQL of child self-report	98.84 ± 3.2	96.72 ± 8.3	0.025^*∗*^
Social score of PedsQL of child's parent report	99.38 ± 2.4	97.41 ± 6.8	0.009^*∗*^
School score of PedsQL of child self-report	95.19 ± 7.5	71.90 ± 24.9	<0.001^*∗*^
School score of PedsQL of child's parent report	95.04 ± 7.2	74.14 ± 28.6	<0.001^*∗*^

CPAP, chronic postappendectomy pain; PedsQL, Pediatric Quality of Life Scale; SD, standard deviation; *p*, statistical error. ^*∗*^Statistical significant. The scores are presented as mean ± SD.

## Data Availability

The data used to support the findings of this study are available in special computers and hospital archive and are available from the corresponding author upon request.
